# Supplementary material for: Intracellular Burkholderia Symbionts induce extracellular secondary infections; driving diverse host outcomes that vary by genotype and environment
Source: ISME J. 2019 Apr 24;13(8):2068–81. doi: 10.1038/s41396-019-0419-7 (PMC6776111; doi:10.1038/s41396-019-0419-7)
Supplement: Supplementary file 1 — Supplementary Table and Figure Legends [file 41396_2019_419_MOESM1_ESM.docx]

**Supplementary Table and Figure Legends:**

**Supplementary Table 1: Organisms used in this study.** Description of bacterial and amoebae isolates used in this study.

**Supplementary Figure 1: Phylogeny of Burkholderia symbionts.** Phylogeny of *D. discoideum Burkholderia* symbionts, and selected other *Burkholderia* from the Plant Beneficial and Environmental clade of *Burkholderia*. Information on the known functions of the different *Burkholderia* species is based on Suarez-Moreno et al. 2012. This multilocus sequence phylogeny is adapted from Haselkorn et al. 2018. Briefly, this maximum likelihood tree (with 1000 bootstrap replicates) was reconstructed based on 3,359bp of DNA from five different housekeeping loci. *Burkholderia mallei* from the pathogenic clade was used as an outgroup.

**Supplementary Figure 2: Similar co-infection patterns are observed when *Rhizobium-*GFP is the sole food source.** Representative confocal micrographs of *Burkholderia*-RFP infected sori contents after development on *Rhizobium*-GFP-only conditions. Similar to 50/50 *Rhizobium*-GFP/*K. pneumoniae* conditions, *Rhizobium* co-infections are only readily visualized for *B. agricolaris* infected sori. Scale bar= 10um.

**Supplementary Figure 3:** **Co-infection patterns persist for Burkholderia and Rhizobium-GFP after host development on food scarce conditions.** Confocal micrographs of *Burkholderia*-RFP infected sori contents after transfer from 50/50 *Rhizobium*-GFP/*K. pneumoniae* conditions and subsequent growth on nutrient scarce conditions. Scale bar= 10um.
