# Supplementary material for: Intracellular Burkholderia Symbionts induce extracellular secondary infections; driving diverse host outcomes that vary by genotype and environment
Source: ISME J. 2019 Apr 24;13(8):2068–81. doi: 10.1038/s41396-019-0419-7 (PMC6776111; doi:10.1038/s41396-019-0419-7)
Supplement: Supplementary file 2 — Supplementary Table 1 [file 41396_2019_419_MOESM2_ESM.docx]

| **Species** | **Strain Name/Paper designation** | **Description** |
| --- | --- | --- |
| *Dictyostelium discoideum* | QS864 | Originally symbiont free wild isolate from the Strassmann-Queller lab originating from Virginia Mt. Lake Biological Station soil. |
| *Burkholderia agricolaris* | Ba70-RFP | Baqs70 symbiont isolated from *D. discodeum* QS70 from the Strassmann-Queller lab originating from Texas Houston Arboretum soil. pmini-Tn7-gat-P1-rfp labeled. |
| *B. agricolaris* | Ba159-RFP | Baqs159 symbiont isolated from *D. discodeum* QS159 from the Strassmann-Queller lab originating from Virginia Mt. Lake Biological Station soil. pmini-Tn7-gat-P1-rfp labeled. |
| *B. agricolaris* | Ba161-RFP | Baqs161 symbiont isolated from *D. discodeum* QS161 from the Strassmann-Queller lab originating from Virginia Mt. Lake Biological Station soil. pmini-Tn7-gat-P1-rfp labeled. |
| *Burkholderia hayleyella* | Bh11-RFP | Bhqs11 symbiont isolated from *D. discodeum* QS11 from the Strassmann-Queller lab originating from Virginia Mt. Lake Biological Station soil. pmini-Tn7-gat-P1-rfp labeled. |
| *B. hayleyella* | Bh171-RFP | Bhqs171 symbiont isolated from *D. discodeum* QS171 from the Strassmann-Queller lab originating from Minisota soil. pmini-Tn7-gat-P1-rfp labeled. |
| *B. hayleyella* | Bh530-RFP | Bh530 symbiont isolated from *D. discodeum* QS530 from the Strassmann-Queller lab originating from Indiana Bloomington soil. pmini-Tn7-gat-P1-rfp labeled. |
| *Burkholderia bonniea* | Bb859-RFP | Bbqs859 symbiont isolated from *D. discodeum* QS859 from the Strassmann-Queller lab originating from Virginia Mt. Lake Biological Station soil. pmini-Tn7-gat-P1-rfp labeled. |
| *B. bonniea* | Bb433-RFP | Bbqs433 symbiont isolated from *D. discodeum* QS433 from the Strassmann-Queller lab originating from Virginia Mt. Lake Biological Station soil. pmini-Tn7-gat-P1-rfp labeled. |
| *B. bonniea* | Bb395-RFP | Bbqs395 symbiont isolated from *D. discodeum* QS395 from the Strassmann-Queller lab originating from Virginia Mt. Lake Biological Station soil. pmini-Tn7-gat-P1-rfp labeled. |
| *Klebsiella pneumoniae* | KpQS1/ Kleb | Food strain from Strassmann Queller lab and Dictybase |
| *Klebsiella pneumoniae* | KpQS1-GFP/ Kleb-GFP | GFP labeled version of above. Pmini-Tn7-KS-GFP |
| *Pseudomonas aeruginosa* | PAO1-GFP/ Pseu-GFP | GFP labeled derivitive of ATCC15692 (Inglis et al., Proc Biol Sci. 2016) (38) |
| *Agrobacterium tumefaciens* | A136-GFP/ Agro-GFP | pmini-Tn7-gat-P1-rfp labeled. reclassified *Rhizobium radiobacter* 51350 in ATCC |
| *Serratia* | Serr-GFP | Isolated from Dictyostelium fruiting bodies grown directly from Virginia Mountain Lake Biological Station soil (42). pmini-Tn7-J=KS-GFP labeled. 16S rRNA sequence identified: GenBank MH997561 |
| *Rhizobium* | Rhiz-GFP | Isolated from Dictyostelium fruiting bodies grown directly from Virginia Mountain Lake Biological Station soil (42). pmini-Tn7-J=KS-GFP labeled. 16S rRNA sequence identified: GenBank MH997560 |
