# Supplementary figures and images for: Intracellular Burkholderia Symbionts induce extracellular secondary infections; driving diverse host outcomes that vary by genotype and environment
Source: ISME J. 2019 Apr 24;13(8):2068–81. doi: 10.1038/s41396-019-0419-7 (PMC6776111; doi:10.1038/s41396-019-0419-7)

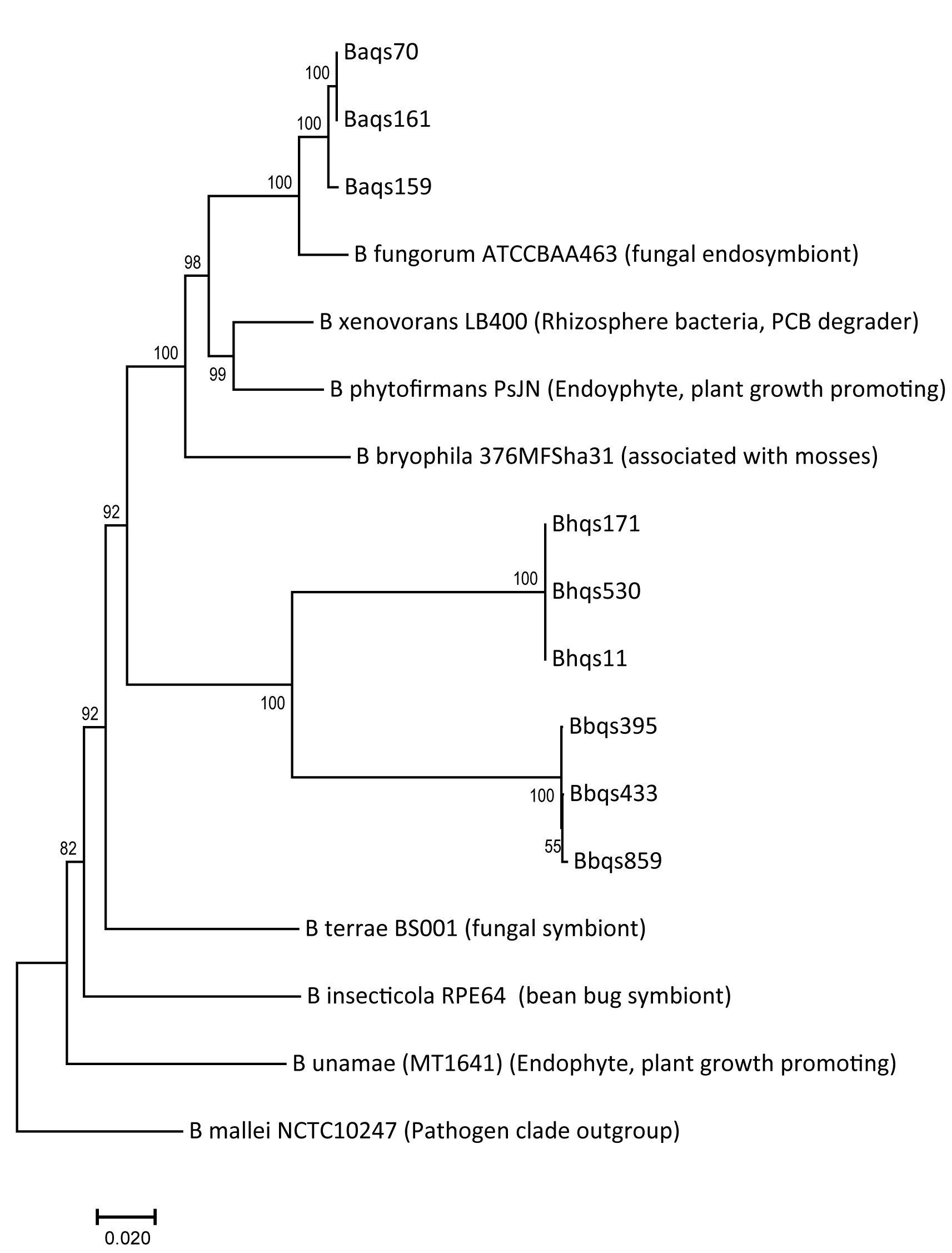

Supplement: Supplementary file 3 — Supplementary Figure 1 [file 41396_2019_419_MOESM3_ESM.tif]

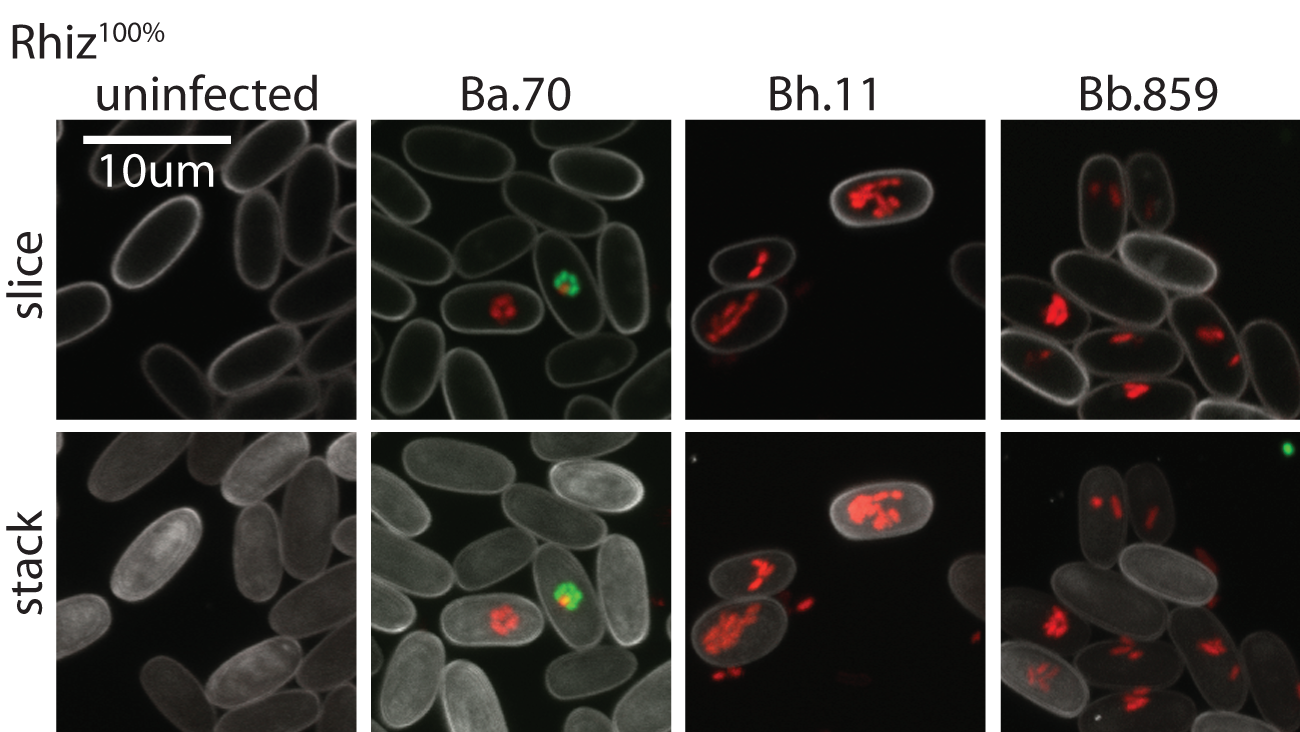

Supplement: Supplementary file 4 — Supplementary Figure 2 [file 41396_2019_419_MOESM4_ESM.tif]

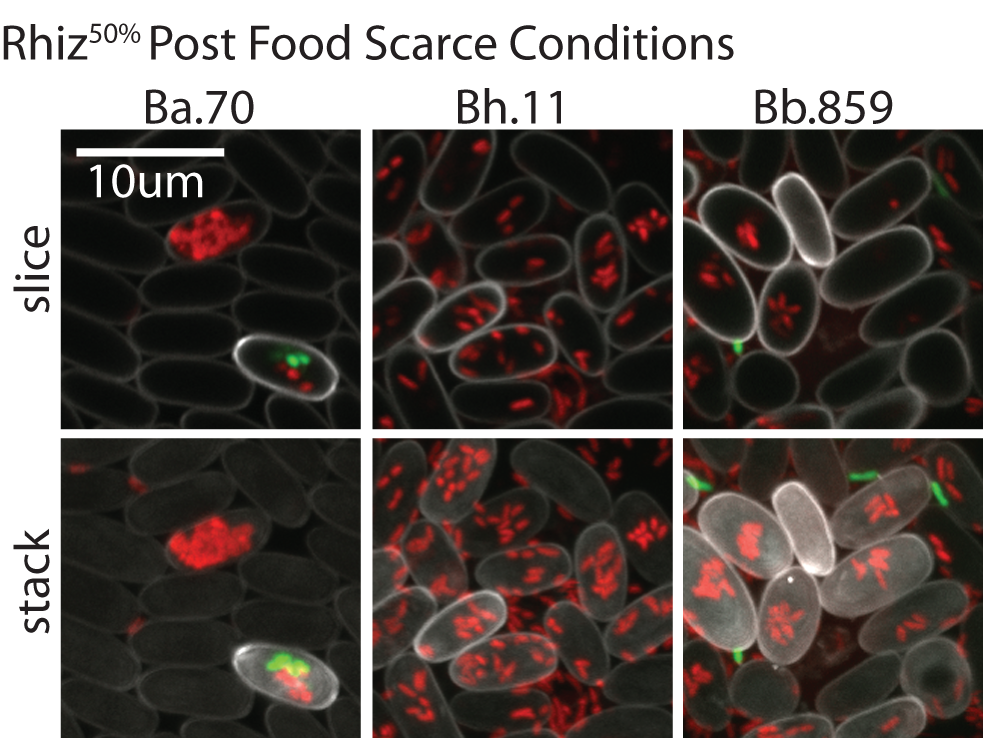

Supplement: Supplementary file 5 — Supplementary Figure 3 [file 41396_2019_419_MOESM5_ESM.tif]
